# Characteristics of multi-institutional health sciences education research: a systematic review

**DOI:** 10.5195/jmla.2017.134

**Published:** 2017-10-01

**Authors:** Jocelyn Huang Schiller, Gary L. Beck Dallaghan, Terry Kind, Heather McLauchlan, Joseph Gigante, Sherilyn Smith

## Abstract

**Objectives:**

Multi-institutional research increases the generalizability of research findings. However, little is known about characteristics of collaborations across institutions in health sciences education research. Using a systematic review process, the authors describe characteristics of published, peer-reviewed multi-institutional health sciences education research to inform educators who are considering such projects.

**Methods:**

Two medical librarians searched MEDLINE, the Education Resources Information Center (ERIC), EMBASE, and CINAHL databases for English-language studies published between 2004 and 2013 using keyword terms related to multi-institutional systems and health sciences education. Teams of two authors reviewed each study and resolved coding discrepancies through consensus. Collected data points included funding, research network involvement, author characteristics, learner characteristics, and research methods. Data were analyzed using descriptive statistics.

**Results:**

One hundred eighteen of 310 articles met inclusion criteria. Sixty-three (53%) studies received external and/or internal financial support (87% listed external funding, 37% listed internal funding). Forty-five funded studies involved graduate medical education programs. Twenty (17%) studies involved a research or education network. Eighty-five (89%) publications listed an author with a master’s degree or doctoral degree. Ninety-two (78%) studies were descriptive, whereas 26 studies (22%) were experimental. The reported study outcomes were changes in student attitude (38%; n=44), knowledge (26%; n=31), or skill assessment (23%; n=27), as well as patient outcomes (9%; n=11).

**Conclusions:**

Multi-institutional descriptive studies reporting knowledge or attitude outcomes are highly published. Our findings indicate that funding resources are not essential to successfully undertake multi-institutional projects. Funded studies were more likely to originate from graduate medical or nursing programs.

## INTRODUCTION

Health sciences educators seek evidence-based teaching approaches to optimize learning outcomes [1]. Stakeholders in education, however, have maintained that the quality of health sciences education research is inadequate [2, 3]. In response to this critique, journal editors and education researchers expect greater methodological rigor, larger sample sizes, and more meaningful outcomes [4–8]. Applicability is increased when studies are generalizable beyond a given teacher, learner, or setting. In health care, multi-institutional research is the cornerstone of clinical trials of treatment and diagnostic innovations for patient care. Because of the larger sample size and more diverse population in multi-institutional clinical research, results may be generalized to a broader population. Therefore, to enhance the generalizability of health sciences education studies and broaden impact, individuals and institutions should also collaborate and conduct multi-institutional health sciences education research [9, 10].

A few publications provide general tips for conducting collaborative research in medical education and include advice on planning, implementation, and dissemination of outcomes [9, 11, 12]. Although helpful, these suggestions are based on the authors’ experiences and are not linked to publication data [13]. In addition, to our knowledge, the types of studies that are most amenable to multi-institutional education research, characteristics of the authors, and the level of support needed for multi-institutional health sciences education research have not been described.

In this systematic review, our initial objective was to collect data on published multi-institutional medical education research to identify common characteristics of these collaborative projects. After consultation with library experts, we used additional search terms, which broadened the scope to capture other health sciences professions publications. Through this review, we sought to inform educators about attributes of published peer-reviewed, multi-institutional health sciences education research as they undertake such projects.

## METHODS

This review was planned and conducted according to Preferred Reporting Items for Systematic Reviews and Meta-Analyses (PRISMA) guidelines [14].

### Eligibility criteria

We included English-language empirical studies from 2004 to 2013 with participants who were in undergraduate or graduate health sciences training programs. Studies were included if they reported educational outcomes (i.e., attitudes, knowledge, or skills) or changes in patient outcomes and were conducted at more than one institution. Publications were excluded if they solely involved faculty development or continuing medical education for practicing professionals. Publications were also excluded if they involved a single training program, even if the trainees rotated at multiple hospital systems.

### Information sources

MEDLINE, the Education Resources Information Center (ERIC), EMBASE, and CINAHL databases were searched.

### Search strategy

We searched for studies using search terms and key words related to (1) multi-institutional; (2) medical education, medical students, graduate medical education, allied health, health occupations, or nursing students; and (3) teaching, education, curriculum, competency, or simulation. Two research librarians independently developed the search criteria with similar results. The search was conducted by Vanderbilt University. The supplemental appendix provides the complete search strategies for each database.

### Study selection

“Health science education research” was defined as any original research study pertaining to health professional students or postgraduate residents and fellows in medicine, nursing, dentistry, or pharmacy. We defined “multi-institutional” as any project that included participants from more than one school or institution. “Original research” was defined as an educational intervention or trial, curriculum evaluation with subjective or objective outcomes, or evaluation of an educational instrument or tool. We included studies that were qualitative and/or quantitative with descriptive and/or experimental research methodologies.

### Data collection and process

**Figure 1:**
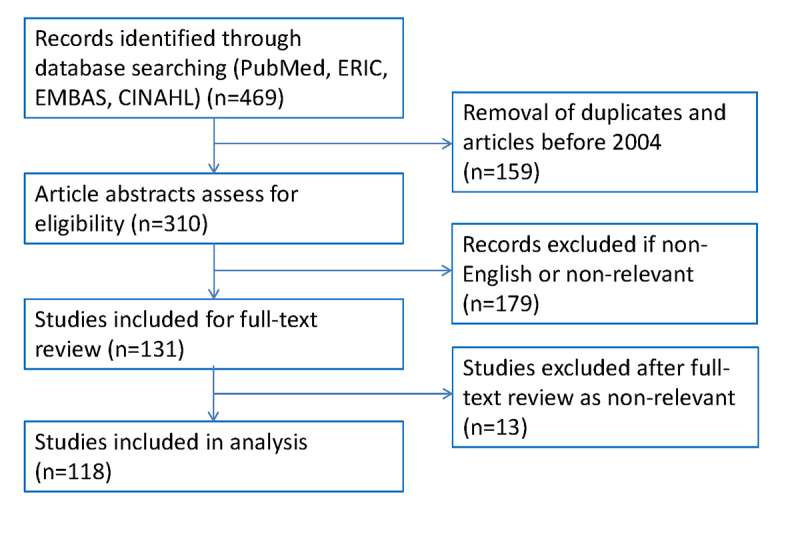
Flowchart of literature search and study selection for multi-institutional health sciences education research

Initially, 469 records were identified (Figure 1). Duplicates were removed, and results were limited to the years 2004–2013, yielding 310 remaining records. These 310 studies were divided among pairs of researchers who independently reviewed the studies’ abstracts to determine whether the publication met our definition of multi-institutional health sciences educational research. This resulted in 131 studies for full review.

We developed and piloted a standardized data abstraction form in Microsoft Excel to document the number of learners in the study, learner level of training, learner field of study, number of institutions of authors and learners, number of authors, author degrees, institutional nationality of learners, external and internal research funding, research methods, and research or educational network involvement. In addition, author affiliations were examined to identify whether a researcher in a department of education participated in the study. To pilot the data abstraction form, each investigator reviewed five publications. A conference call was held to reach consensus on the results as well as to better define categories for consistency of data extraction.

Three pairs of authors then reviewed the remaining 131 articles (divided per pair). Discrepancies in coding were resolved by each pair of authors or brought to the larger research team for consensus. After full review, an additional 13 articles did not meet the initial inclusion criteria, leaving 118 publications for final analysis. One pair of authors reviewed all publications to determine study type (experimental or descriptive) and outcomes. If multiple outcomes were examined, studies were categorized according to the “highest” domain of educational activity, assessed using a modification of Kirkpatrick’s model: (1) learner reaction and attitude, (2) acquisition of knowledge, (3) demonstration of skill, and (4) changes in patient care [15, 16].

### Data analysis

Descriptive and correlational statistics were used to summarize the findings of the systematic review using IBM SPSS, version 22, software.

## RESULTS

The average number of learners across studies was 379 (median 188; range 4–4,300) from an average of 8.4 institutions (median 5; range 2–73). Most studies included participants in graduate medical education programs (60%; n=71), followed by medical students (38%; n=45) and nursing students (11%; n=13). Sixteen percent (n=19) of studies included multiple levels of learners, but only 2 were interdisciplinary, with the remainder involving a combination of medical students and residents or undergraduate and graduate nursing students. Of the 89 studies involving medical students or residents, 44% (n=39) involved surgical and 22% (n=20) involved internal medicine departments.

Most learners were from institutions in the United States (69%; n=82), followed by Canada (7%; n=8). The publications in this analysis included learners from 26 countries; 8% (n=9) included learners from more than 1 country.

The median number of authors was 7 (range 1–20). Of studies published in journals noting author degrees (81%; n=96), the majority listed an author with a master’s degree or doctorate (PhD) (89%; n=85). A minority of publications noted an author in a Department of Medical Education (18%; n=21) or Department of Biostatistics or Epidemiology (25%; n=29). Seventeen percent (n=20) of studies acknowledged an affiliation with a network, association, registry, or study group.

**Figure 2:**
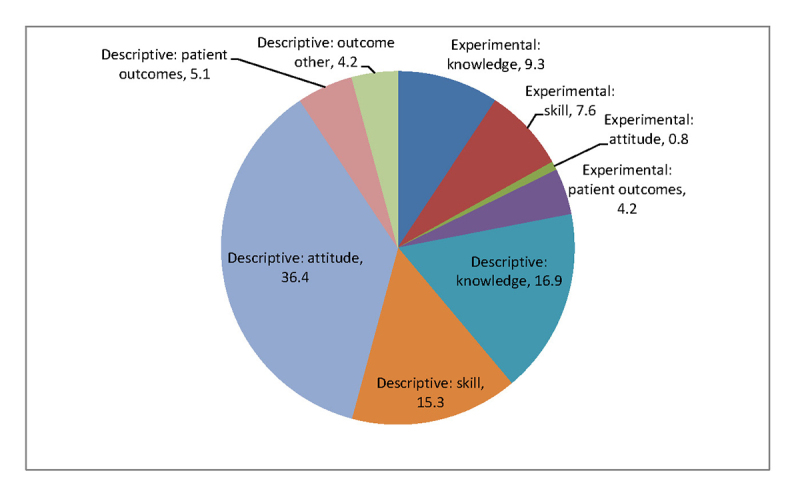
Distribution of study type and outcome measures of the reviewed publications (%)

Study types were heterogeneous. Twenty-six (n=22%) studies used an experimental design, and 16 of these studies randomized learners to different conditions (14% of all studies). Most studies were descriptive (78%; n=92). The study outcomes reported were changes in student attitude (38%; n=44), knowledge (26%; n=31), and skill assessment (23%; n=27), as well as patient outcomes (9%; n=11). Figure 2 shows the distribution of study types and outcome measures of the reviewed publications. Most studies (82%; n=97) used quantitative methods, with the remainder using qualitative methods (10%; n=12) or a mixed methods approach (8%; n=9).

**Figure 3:**
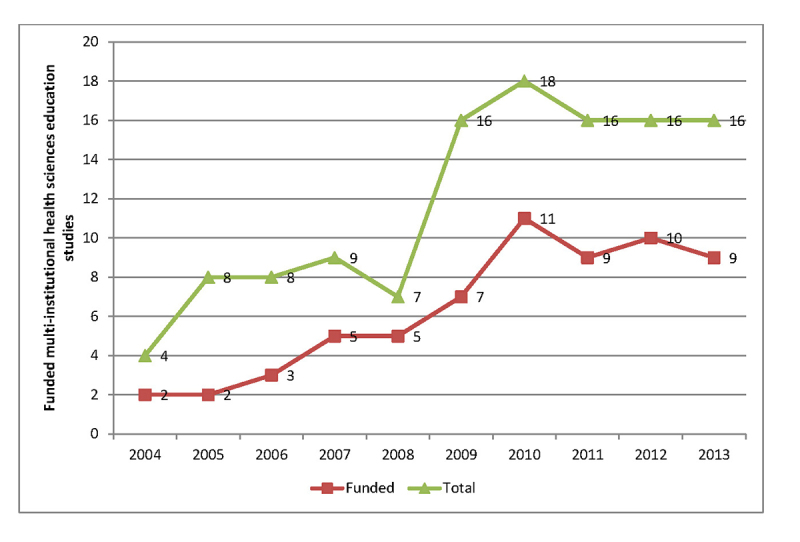
Funding trends of the reviewed publications

Fifty-three percent (n=63) of publications acknowledged funding, with a steady increase in the frequency of funding over time (Figure 3). As the number of multi-institutional health sciences education publications increased, there was a correlating rise in the number of funded studies (r=0.919; *p*<0.001). Of these funded projects, 87% (n=55) received external funding, and 37% (n=23) received internal funding. Of the funded studies, 71% (n=45) were from graduate medical education programs, 41% (n=26) of which were conducted in surgical specialties and 36% (n=22) of which were conducted in primary care specialties (internal medicine, pediatrics, psychiatry, family medicine).

## DISCUSSION

We conducted a structured review of multi-institutional undergraduate and graduate health sciences literature from the past decade to identify characteristics of collaborative projects. Our results indicate that multi-institutional educational research can be successfully carried out and published with limited infrastructure support, but they also point out important opportunities for future work. In our analysis, just over half the studies reported funding, and fewer than 20% reported involvement in a network or collaborative organization.

Funding is thought to enhance health sciences education research by facilitating support of rigorous study designs through multi-institutional collaboration [2]. Multi-institutional collaboration necessitates deliberate, prospective research designs in order to investigate interventions of comparative settings [17]. Although rigor based on Reed and colleagues’ recommendations [2] can lead to improved funding rates, our findings indicate that studies using a variety of study methodologies were also funded. Fifty-three percent of multi-institutional health sciences education research studies in our review reported funding, similar to rates reported in prior studies [18–20].

We found that 45 (75%) funded studies involved residency training programs, 26 of which were from surgical subspecialties. This might be the result of efforts by the American College of Surgeons to support regional simulation-based education [21], which helped residency training programs undertake multi-institutional research related to skills development in surgical residency programs. With funding and technical training as common bridges across institutions [22], residency programs were more likely to be able to support multi-institutional studies due to common requirements through the Accreditation Council for Graduate Medical Education [23]. The American Association of Colleges of Nursing have similar “essential” guidelines for all nursing programs [24], which might explain why multiple nursing studies appeared in our search. Since medical student education can be starkly different across institutions in an era of curricular innovation, congruence of specific disciplinary focus [22] may limit the ability to conduct a rigorous, multi-institutional research study.

Transforming educational activities into high-quality scholarship that advances the field requires methodological skills and resources [9, 11, 25, 26], but most health sciences faculty are not trained in educational or other social scientific research [27]. Investigators with educational research expertise can provide valuable resources to support educational scholarship. The majority of publications included in our study listed an author with a master’s degree or PhD, suggesting advanced training in research methodology. Of funded studies, 64% had authors with such degrees. Due to differences in the publication style of various journals, it was unclear how many authors were from a Department of Medical Education or Department of Biostatistics or Epidemiology or were involved with a research network. Because some journals did not note the credentials of their authors or affiliations, it was possible that the true numbers of authors with advanced degrees or in these departments were higher. Because lack of research expertise has been identified as a major barrier to health sciences education research [26, 28, 29], multi-institutional collaborations and research networks may provide the support needed to overcome these obstacles.

Faculty undertaking future educational research should identify potential resources in their institutions and effectively leverage national programs that support skills development and collaboration [30, 31]. National organizations should continue to invest in infrastructure to support research networks and anticipate the financial needs of their ongoing maintenance and growth [32]. We did find an increase in the number of studies reporting funding over the ten-year time span of this project, perhaps reflecting the acknowledgement of prior calls for increased funding for health sciences education research funding [18, 19, 33, 34].

A minority of the reviewed studies employed experimental methods, consistent with previous findings [19, 35], and fewer than 10% of studies measured patient outcomes despite repeated calls for this focus [36]. Accountability, safety, and quality are pressing needs in health care. Health sciences education research, and more specifically medical education research, must develop rigorous, generalizable outcome measures that guide curricular change to improve the health of patients [5]. Guidance exists for educational researchers to address these quality gaps, which can provide a foundation for designing future studies [37]. Multi-institutional studies, while resource intensive, can add to this effort by improving generalizability of findings. In addition, collaborative research may help facilitate health sciences education researchers and patient outcomes researchers to leverage their skills.

Several limitations of this study should be considered. Though our analysis was limited to a ten-year period and included studies available in the English language only, we included the most recent available decade and included studies from around the world. In using “AND” as well as “health occupations” in the search strategy, we may have excluded some studies in professions outside of medicine. However, as our initial objective was to study medical education, we believe the inclusion of other health sciences in this study has broader appeal. Future investigations should specifically include other health professions by name to draw an even more comprehensive picture. We defined success as publication and described characteristics of published studies but did not include or describe characteristics of unpublished multi-institutional studies. We also did not examine single-institutional studies for comparison.

In this systematic review, we describe the current state of multi-institutional health sciences education publications to assist educators who are planning to undertake such collaborative projects. Collaboration can assist in planning for resources, developing research networks, and creating infrastructure whether regionally, nationally, or internationally within or across specialty societies. Most study teams collaborated with a team member with a PhD or master’s degree, and more than half had funding for their research. Our results indicate that multi-institutional educational research can be successfully carried out and published with limited infrastructure support. Financial and educational resources to foster and support collaborative educational research may be helpful to promote future high-quality multi-institutional medical education research.

## SUPPLEMENTAL FILE

Appendix Complete search strategies for each database
