# Supplementary material for: Characteristics of multi-institutional health sciences education research: a systematic review
Source: J Med Libr Assoc. 2017 Oct 1;105(4):328–35. doi: 10.5195/jmla.2017.134 (PMC5624422; doi:10.5195/jmla.2017.134)
Supplement: Appendix [file jmla-105-328-s001.pdf]

JMLA Journal of the Medical Library Association

SC

Supplemental content to
J Med Libr Assoc. Oct;105(4): dx.doi.org/10.5195/jmla.2017.134
www.jmla.mlanet.org
© Schiller, Beck Dallaghan, Kind, McLauchlan, Gigante, Smith 2017

Characteristics of multi-institutional health sciences education research: a systematic review

Jocelyn Huang Schiller, MD; Gary L. Beck Dallaghan, PhD; Terry Kind, MD, MPH; Heather McLauchlan, MD; Joseph Gigante, MD; Sherilyn Smith, MD

APPENDIX

Complete search strategies for each database

Vanderbilt University Medical Center
The Annette and Irwin Eskind Biomedical Library
Nashville, TN 37232-8340
Phone: 615.936.1410
Fax: 615.936.1384

Multi-institutional medical education studies
Last updated September 24, 2013

PubMed search strategy

Search terms	Preliminary search results
#1 ("Multi-Institutional Systems"[Mesh] OR "Hospital Shared Services"[Mesh] OR (multi[tiab] AND institutional[tiab]) OR (multi[tiab] AND institution[tiab]) OR "multi institution"[tiab] OR "multi-institution"[tiab] OR "multi hospital systems"[tiab] OR "multihospital system"[tiab] OR "multicenter"[tiab])	68,862
#2 ("education, medical"[MeSH Terms] OR "medical education"[tiab] OR "medical students"[tiab] OR "medical student"[tiab] OR "Students, Health Occupations"[Mesh] OR "Internship and Residency"[tiab] OR "clinical clerkship"[mesh] OR "residents"[tiab] OR "clerkship"[tiab] OR "clerkships"[tiab])	217,572
#3 ("Teaching"[Mesh] OR "teaching rounds"[tiab] OR "Clinical Competence"[Mesh] OR "clinical competence"[tiab] OR "competency-based education"[mesh] OR "education, distance"[mesh] OR "distance education"[tiab] OR "distance learning"[tiab] OR "educational measurement"[mesh] OR "problem-based learning"[mesh] OR "problem-based learning"[tiab] OR "Curriculum"[Mesh] OR "curriculum"[tiab] OR "educational models"[tiab] OR simulation lab[tiab] OR simulation laboratories[tiab] OR simulation laboratory[tiab] OR simulation labs[tiab] OR "simulation learning"[tiab] OR "education"[tiab] OR "USMLE"[tiab] OR "United States Medical Licensing Exam"[tiab])	423,056
#4 case reports[pt] OR letter[pt] OR comment[pt] OR editorial[pt] OR practice guideline[pt] OR historical article[pt] OR review[pt] OR news[pt] OR newspaper article[pt] OR legal cases[pt] OR meta-analysis[pt]	4,882,333
#5 1 AND #2 AND #3 NOT #4	301
#6 #5 AND English[la]	283

Key: [mh] Medical Subject Heading; [tiab] title/abstract word; [la] language; [pt] publication type.

© Schiller, Beck Dallaghan, Kind, McLauchlan, Gigante, Smith 2017

Education Resources Information Center (ERIC) search strategies (Proquest platform) September 20, 2013

Search terms	Preliminary search results
#1 SU.EXACT("Educational Cooperation") OR SU.EXACT("Cooperative Planning") OR SU.EXACT("Consortia") OR SU.EXACT("International Cooperation") OR SU.EXACT("Agency Cooperation") OR SU.EXACT("Shared Resources and Services") OR SU.EXACT("Institutional Cooperation") OR SU.EXACT("Cooperative Programs") OR "interagency" OR "multi-institutional" OR "multiinstitutional"	30,185
#2 (SU.EXACT("Medical Education") OR SU.EXACT("Graduate Medical Education") OR SU.EXACT("Nursing Students") OR SU.EXACT("Allied Health Occupations") OR SU.EXACT("Medical Students") OR "residents" OR "residency" OR "clerkship" OR "clerkships" OR "medical student" OR "medical students" OR "graduate medical students" OR "medical education")	22,087
#3 (SU.EXACT("Internship Programs") OR SU.EXACT("Experiential Learning") OR SU.EXACT("Problem Based Learning") OR SU.EXACT("Field Experience Programs") OR SU.EXACT("Clinical Teaching (Health Professions)") OR SU.EXACT("Simulation") OR SU.EXACT("Classroom Techniques") OR SU.EXACT("Teaching Models") OR SU.EXACT("Clinical Experience") OR "clinical training" OR SU.EXACT("Instruction") OR SU.EXACT("Learning Strategies") OR SU.EXACT("Practicums") OR SU.EXACT("Teaching Methods") OR "simulations" OR SU.EXACT("Course Content") OR SU.EXACT("Curriculum Evaluation") OR SU.EXACT("Integrated Activities") OR SU.EXACT("Technology Integration") OR SU.EXACT("Online Courses") OR SU.EXACT("Models") OR SU.EXACT("Instructional Innovation") OR SU.EXACT("Program Development") OR SU.EXACT("Educational Strategies") OR SU.EXACT("Distance Education") OR SU.EXACT("Blended Learning") OR SU.EXACT("Electronic Learning") OR SU.EXACT("Curriculum Enrichment") OR SU.EXACT("Curriculum Development") OR SU.EXACT("Experimental Teaching") OR SU.EXACT("Curriculum Design") OR SU.EXACT("Educational Development"))	376,237
#4 #1 AND #2 AND #3	195
#5 #4 AND Scholarly journals	53

EMBASE search strategies September 20, 2013

Search terms	Preliminary search results
#1 exp multihospital system/	6,302
#2 exp medical education/	239,024
#3 #1 AND #2	39
#4 #1 AND #2 AND "journal articles"	31

JMLA Journal of the Medical Library Association

SC

Supplemental content to
J Med Libr Assoc. Oct;105(4): dx.doi.org/10.5195/jmla.2017.134
www.jmla.mlanet.org
© Schiller, Beck Dallaghan, Kind, McLauchlan, Gigante, Smith 2017

CINAHL search strategies September 24, 2013

Search terms	Preliminary search results
#1 (MH "Multiinstitutional Systems") OR "multihospital" OR "multiinstitutional" OR "multicenter"	17,524
#2 (MH "Education, Medical+") OR (MH "Education, Health Sciences+") OR "Medical Education" OR "graduate medical education" OR "residency" OR "clerkship" OR "medical students"	169,827
#3 (MH "Teaching+") OR (MH "Education, Clinical+") OR (MH "Curriculum+")	132,671
#4 #1 AND #2 AND #3	102

Total of 469 references.
Used Endnote to delete duplicate records (32 duplicates).
453 references.
